# Evidence for a visuospatial bias in decimal number comparison in adolescents and in adults

**DOI:** 10.1038/s41598-019-51392-6

**Published:** 2019-10-14

**Authors:** Margot Roell, Arnaud Viarouge, Emma Hilscher, Olivier Houdé, Grégoire Borst

**Affiliations:** 1 Université de Paris, LaPsyDÉ, CNRS, F-75005 Paris, France; 2 Ecole des Neurosciences de Paris (ENP), Paris, France; 3 Institut Universitaire de France, Paris, France

**Keywords:** Cognitive neuroscience, Cognitive control

## Abstract

There is a close relation between spatial and numerical representations which can lead to interference as in Piaget’s number conservation task or in the numerical Stroop task. Using a negative priming (NP) paradigm, we investigated whether the interference between spatial and numerical processing extends to more complex arithmetic processing by asking 12 year olds and adults to compare the magnitude of decimal numbers (i.e., the prime) and, subsequently, the length of two lines or the luminance of two circles (i.e., the probe). We found NP effects when participants compare length but not luminance. Our finding suggests that decimal comparison is impacted by a visuospatial bias due to the interference between the magnitude of the numbers to be compared and their physical length. We discuss the educational implications of these findings.

## Introduction

The close relationship between number and space can be demonstrated by the spatially organized representations of numbers left by primitive cultures as early as the third millennium BCE^1^. Such a deep connection is discernible in the human brain^2,3^. Indeed, the neural circuitry crucial for numerical representations is located in the parietal lobe in regions that overlap with the neural circuitry involved in spatial representations^4^. Simon *et al*.^5^ observed that calculation activates the intra-parietal sulcus (IPS) in the parietal lobe close to areas activated in visuospatial tasks, such as eye saccades, spatial attention and pointing. Additionally, Harvey and colleagues^6^ demonstrated that the human parietal cortex hosts overlapping, topographically organized maps for both object size and number.

The overlap observed between areas in the parietal cortex coding for space and number could partly explain why spatial processing interferes with numerical processing. A seminal example of such interference can be observed in Piaget’s number-conservation task, in which children up to 7 years of age commit systematic errors in judging the numerical equivalence of two rows of tokens when they differ in length^7–9^. Studies have provided converging evidence that adults and 10-year-old children need to inhibit the length of the rows to compare the number of tokens in each of them to succeed in Piaget’s number-conservation task^7,8,10^. Dormal and Pesenti^11^ reported similar interference between length and numerical processing using a non-symbolic numerical Stroop task in which adult participants were asked to compare either the number of dots or the length of arrays of dots. When asked to compare the number of dots in the arrays, participants were slower and made more errors when the longest array had the smallest number of dots compared to when the longest array had the greatest number of dots. Interference between space and numerical processing is not limited to non-symbolic numerical processing, as evidenced by the size-number congruity effect in a symbolic number Stroop task^12^. Indeed, participants require more time to compare (a) the magnitude of two whole numbers when the greater number is presented in a smaller font than the smaller number^13,14^ and (b) the physical size font of two numbers when the size font is greater for the smaller number than for the greater number^15,16^.

A functional Magnetic Resonance Imaging (fMRI) study provided evidence that the privileged relation between size and number representations could be rooted in the overlap between the activations elicited by number and size representations in the IPS^17^. In this study, participants were asked to perform three comparison tasks: a symbolic number comparison task in which participants compared the magnitude of two Arabic digits, a size comparison task in which they compared the size of two digits, and a luminance comparison task in which they compared the luminance of two digits. The same items were presented in all three comparison tasks, i.e., two digits were displayed on the screen that vary in numerical magnitude, physical size and luminance (e.g., 2 7), and participants were asked to focus on one of the three dimensions (i.e., number, size or luminance). Thus, in certain items, one (or two) of the continuous quantities could interfere with the comparison to perform on the third continuous quantity. Hence, the interference between one dimension and the others could be estimated. Pinel *et al*.^17^ provided evidence of an interference (a) between size and number processing, (b) between size and luminance processing but not between luminance and number processing. In addition, the extent to which these three dimensions (i.e., number, size and luminance) interfered with each other was related to the degree of overlap of the brain activations elicited by these dimensions: activations elicited by number and size dimensions overlapped in the anterior horizontal segment of the IPS, while the activations elicited by size and luminance overlapped in the posterior part of the IPS and the ventral occipito-temporal cortex. Importantly, no overlap of activation was found for the luminance and number dimensions. Pinel and colleagues’^17^ findings point toward the existence of distinct but overlapping systems of representations of numerical and non-numerical dimensions of magnitude (see also^18,19^) with more overlap between the number and space dimensions than between number and luminance (but see^20^). Similarly, Kucian and colleagues^21^ examined the development of the association between discrete non-symbolic numerical processing, namely dot arrays, and non-numerical continuous processing, specifically angles. They found that magnitude processing of dot arrays and angles were positively related to each other. Importantly, as they found that the developmental patterns of number and space processing were different, the authors suggested that non-symbolic numerical processing and continuous spatial processing were operated by dissociated but closely related magnitude systems^21^.

Behavioral studies provided converging evidence for the selective interference between the numerical and the space dimensions^22–25^. Viarouge & de Hevia^25^ showed that participants overestimated the size of a square to a greater extent when delimited by large (e.g., 8 or 9) than when delimited by small (e.g., 1 or 2) numbers, but that they did not display such bias when estimating the luminance of a square flanked by small or large numbers. Patro, Nuerk, Cress and Haman^26^ proposed a taxonomy of spatial-numerical associations in young children in which they distinguish between cross-dimensional magnitude processing, association between spatial and numerical intervals, association between cardinalities and associations between ordinalities and spatial directions. In the present study, we examined a case of cross-dimensional magnitude processing. One possible explanation for the privileged relation between spatial and numerical processing could be that dedicated neural circuits for processing non-numerical dimensions of magnitude such as space but not luminance are co-opted during the acquisition of complex mathematical skills such as symbolic number processing^27^.

In the present study in adolescents (i.e., 12-year-olds) and young adults (i.e., 20-year-olds), we investigated whether the interference between space and numerical processing extend to more complex arithmetic processing, such as comparing decimal numbers. We focused on decimal number comparison, as children and even educated adults have difficulty in comparing decimal numbers when the smallest decimal number has the greatest number of digits (e.g., 0.6 vs. 0.131)^28–32^. So far, studies have mainly attributed this difficulty to a whole number bias, i.e., an overgeneralization of whole number properties to rational numbers^30,32–36^. The whole number bias consists in using a property of whole numbers, such as “the greater the number of digits, the greater its magnitude”, to compare decimal numbers in which the smallest one has the greatest number of digits after the decimal point (e.g., 0.6 vs. 0.131)^32,36,37^. However, the comparison of the magnitude of two decimal numbers in which the smallest decimal number has the greatest number of digits (e.g., 0.6 vs. 0.131) might rely not only on the inhibition of the property of whole numbers such as the “greater the number of digits, the greater its magnitude,” (see^35^ for evidence in both adolescents and adults) but also on the inhibition of the physical lengths of the decimal numbers per se.

Therefore, we investigated more specifically in the present study (a) whether comparing the magnitude of two decimal numbers in which the smallest one has the greater number of digits after the decimal point and thus is the longest one (e.g., 0.6 vs. 0.131) requires one to inhibit non-numerical continuous dimensions, such as the lengths of the decimal numbers, not only in 12-year-old adolescents but also still in adults, and (b) whether the inhibition of non-numerical continuous dimensions when comparing decimal numbers is specific to space, consistent with the functional overlap reviewed above, or extends to other non-numerical continuous dimensions such as luminance. To do so, we designed a negative priming (NP) task based on our previous studies^34,35^. The NP paradigm rests on the rationale that if a strategy (or distractor) is inhibited on a given item, then the activation of that strategy (or distractor) on the next item should be more difficult, as revealed by poorer performance^38^. In the present study, adolescents and adults performed a negative priming task in which they were asked to compare the magnitude of two decimal numbers, the length of two lines or the luminance of two circles. It is important to note that we selected circles to present the luminance stimuli in order to separate the luminance dimension from the spatial dimension. Each trial consisted of a prime item followed by a probe item^39^. Participants compared, on the prime item, the magnitude of two decimal numbers and later, on the probe item, the length of two lines or the luminance of two circles. In the test condition, the probe items were preceded by a pair of decimal numbers in which the smallest number had the greatest number of digits after the decimal point and thus was the longest (e.g., 0.6 vs. 0.131, a context in which the length of the decimal numbers interferes with their magnitude). In the control condition, the probe items were preceded by a pair of decimal numbers with the same number of decimal places and thus the same length (e.g., 0.612 vs. 0.157, a context in which length is neither congruent nor incongruent with the magnitude of the decimal numbers to be compared).

We reasoned that if the lengths of the decimal numbers must be inhibited at both ages to compare the magnitude of two decimal numbers in which the smallest number is the longest one, adolescents and adults should be less efficient in comparing the length of two lines when these are preceded by a pair of decimal numbers in which the smallest one has more digits after the decimal point and thus is the longest one (e.g., 0.6 vs. 0.131) than when preceded by a pair of decimal numbers with the same number of digits after the decimal point and thus are the same length (e.g., 0.612 vs. 0.157)^34^. In addition, if the inhibition of the non-pertinent non-numerical continuous dimension is specific to the lengths of the numbers, in line with the functional overlap between the areas that process number and space in the IPS^17,40^, we expect a negative priming effect only when the probes require a length comparison but not when they require a luminance comparison.

Testing both adolescents and adults allows us to examine (a) whether educated adults must still inhibit the lengths of the decimal numbers to process their magnitude comparing two decimal numbers which have more digits after the decimal point and thus are the longest (0.6 vs. 0.131) and (b) whether the efficiency to inhibit the non-pertinent non-numerical continuous dimension (i.e., the length of the decimal number) to process the pertinent one (i.e., the magnitude of the decimal number) increases with age in this context. Consistent with previous findings^41,42^, we predicted the amplitude of the negative priming effect to be larger in adolescents than in adults. To control that any difference in negative priming amplitude between adolescents and adults was not due to differences in general inhibitory control efficiency, participants performed a seminal inhibitory control task, the Color-Word Stroop task (see Supplementary Materials)^43^.

## Materials and Methods

### Participants

We recruited 48 university students (mean age = 20.0 ± 1.69 years) with normal or corrected-to-normal vision (14 males and 34 females). All adults received course credits for their participation and provided informed written consent.

We also recruited 62 adolescents in 7th grade (mean age = 12.2 ± 0.45 years) with normal or corrected-to-normal vision from a public high-school serving a diverse population. We excluded eleven adolescents who scored at the chance level of performance on the decimal comparison NP task, leading to a group of 51 adolescents (29 males and 22 females, mean age = 12.27 ± 0.45 years). We obtained informed written consent from parents as well as oral consent from all adolescents. The proportion of males-to-females was found to be significantly different between the two groups, _*X*_^2^(1) = 7.72, p = 0.005; we therefore included the variable ‘gender’ in our models.

We ran an a priori power analysis using G * Power 3.1.9.2^44^ to determine the sample size. The power analyses revealed that a minimum of 82 participants would be needed to detect a medium effect size of 0.25 (according to Cohen’s effect size conventions) within a 2 (Condition: control vs. test) × 2 (Block: Line vs. Luminance) × 2 (Age: Adolescent vs. Adult) mixed-design ANOVA, with a power (1 - β) set at 0.80 and a α set at 0.05.

The internal ethical board of the Faculty of Psychology (Paris Descartes University) ruled that in light of the potential risks for the participants of the present study, no formal ethical approval by one of the national ethical committee was needed in agreement with the Ethical law governing human research in France. All participants were tested in accordance with national and international norms governing the use of human research participants.

### Materials and procedures

Stimuli were presented on a laptop computer (screen resolution of 1366 × 768 pixels with a refresh rate of 60 Hz), using E-Prime 2.0. All the participants first performed the decimal comparison NP task followed by the Color-Word Stroop task (See Supplementary Materials). The experimental session lasted approximately 30 minutes per participants. Participants were tested individually and seated approximately 75 cm from the laptop computer.

We designed our negative priming task based on our previous studies^34,35^. Three types of pairs of stimuli with decimal numbers, lines and circles were created. Sixty pairs of decimal numbers were composed of two decimal numbers written in 24-point Courier New font, each located at the left or right side of the screen at a 1° visual angle from the center. In 24 of the pairs of decimal numbers, the smallest decimal number had the greatest number of digits after the decimal point and thus was the longest one (e.g., 0.6 vs. 0.131). In 24 of the pairs, the two decimal numbers had the same number of digits after the decimal point and thus the same length (e.g., 0.612 vs. 0.157). In each of the two types of pairs, one-third of the decimal numbers had one digit after the decimal point, one-third two digits and one-third three digits. We also included twelve pairs of decimal numbers in which the largest decimal number had the greatest number of digits after the decimal point (e.g., 0.642 vs. 0.18) to prevent participants from systematically choosing the smallest decimal number when the length of the two decimal numbers differed. The average numerical distance between the two decimal numbers was the same in the three types of pairs (M = 0.29 ± 0.20 vs. M = 0.27 ± 0.20 vs. M = 0.33 ± 0.21, F < 1), see Supplementary Table S1 for the full list of decimal number pairs. In half of the three types of decimal number pairs, the largest decimal number was displayed on the right side of the screen.

Sixty pairs of lines were composed of two lines of different lengths, one displayed at the right side and one at the left side of the screen at random locations. Five different length ratios were used (1.25, 1.1, 1, 0.73 and 0.8) with three different levels of lengths (10, 12 and 15 cm), for a total of 12 different pairs. Lengths ranged from 7.29 cm to 18.75 cm (with a visual angle ranging from 4.24° to 10.8°) across all 12 pairs. Lines were positioned at 1° of visual angle from the center of the screen. In half of the pairs the longest line was displayed at the right side of the screen. Sixty pairs of circles were composed of two circles of different luminance (with a visual angle of 4°): one displayed at the right side and one displayed at the left side of the screen. Five different luminance ratios were used (1.4, 1.2, 1, 0.6, and 0.8) with three levels of luminance (defined by shades of gray with identical RGB values 100, 150, and 125), for a total of 12 different pairs. Circles were positioned at 1° of visual angle from the center of the screen. Luminance ranged from RGB value 60 to 210 across all 12 pairs. In half of the pairs, the brightest circle was displayed at the right of the screen.

**Figure 1:**
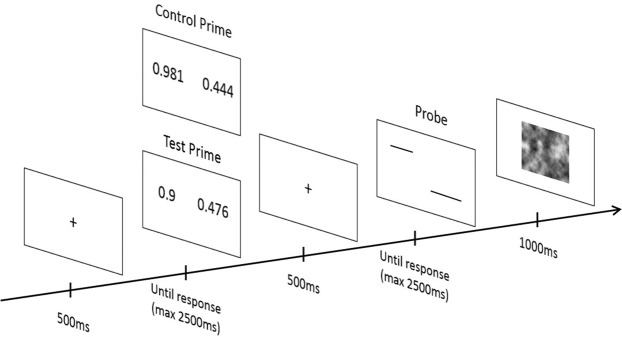
Example of prime and probe items presented in the test and the control conditions of the Decimal Comparison NP task’s Line Block. Prime items (i.e., incongruent and neutral items) differed between the two conditions but the probe items were similar.

**Figure 2:**
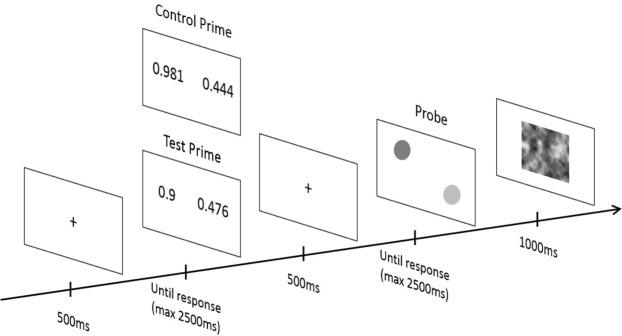
Example of prime and probe items presented in the test and the control conditions of the Decimal Comparison NP task’s Luminance Block. Prime items (i.e., incongruent and neutral items) differed between the two conditions but the probe items were similar.

As shown in Figs 1 and 2, each trial began with the presentation of a fixation cross (500 ms), and a pair of decimal numbers (i.e., the prime) was displayed until the participant answered (with a time limit of 2500 ms). Participants pressed the left or right button of the mouse to indicate that the decimal number presented at the left or the right side of the screen was the largest. As soon as the participant provided an answer the fixation cross reappeared (500 ms) followed by a pair of lines (in the Line Block) or a pair of circles (in the Luminance Block) (i.e., the probe), which persisted until the participant provided an answer (with a time limit of 2500 ms). Participants pressed the left or right button of the mouse to indicate that the line or the circle presented at the left or the right side of the screen was the longest (for the line) or the brightest (for the circle). A visual mask was presented between each trial to avoid the transfer of processes from the probe of one trial to the prime of the next trial (1000 ms).

Participants performed two experimental blocks (Line Bock and Luminance Block) of 60 trials: 24 control trials, 24 test trials and 12 filler trials. The order of the Line and Luminance Block was counterbalanced between participants (half the participants began with the Line Block and half with the Luminance Block). In both Blocks, control, test and filler trials differed only with respect to the type of decimal number pairs displayed on the prime. In the control trials, pairs of decimal numbers with the same number of digits after the decimal point (0.612 vs. 0.157) were presented on the prime. In the test trials, pairs of decimal numbers in which the smallest decimal number had the greatest number of digits after the decimal point (0.6 vs. 0.131) were presented on the prime. Lastly, in the filler trials, pairs of decimal numbers in which the largest one had the greatest number of digits after the decimal point (0.642 vs. 0.18) were presented on the prime. All sequences of motor responses between the prime and the probe appeared equally often. The order of the trials was pseudo-randomized, with no more than three tests or control trials occurring in a row. Before each experimental block, participants performed 6 training trials (using stimuli different from the ones used in the experimental trials) with pairs of decimals and pairs of lines (in the Line Block) or pairs of decimals and pairs of circles (in the Luminance Block). During the training trials, participants received simple feedback on accuracy (correct/incorrect).

## Results

For each participant, we averaged the RTs and accuracy rates separately for the control and the test primes and probes. Prime or probe responses times (RTs) +/− 2 SD from the individual mean for a given condition were deleted. As a result, on average, 5% (±1.21%) and 5% (±1.11%) of the RTs were deleted for adolescents and adults respectively. We computed, for each participant, the inverse efficiency score, IES (i.e., RTs divided by the proportion of correct answers, see^45^) for the primes and the probes of the control and the test conditions. Importantly, our data complied with the recommendations of Bruyer and Brysbaert^45^ for the use of IES: accuracy was high (i.e., mean greater than 98.5%), accuracies and RTs went in the same direction and we observed no speed-accuracy trade-off (*r*s < 0.17, *p*s > 0.09). Note that, in accordance with the logic of the NP paradigm, we computed the IES by including RTs on prime items performed accurately (on average 96.5% of the trials) and on probe items performed correctly preceded by prime items performed accurately (on average 95.4% of the trials, see Supplementary Table 2).

**Figure 3:**
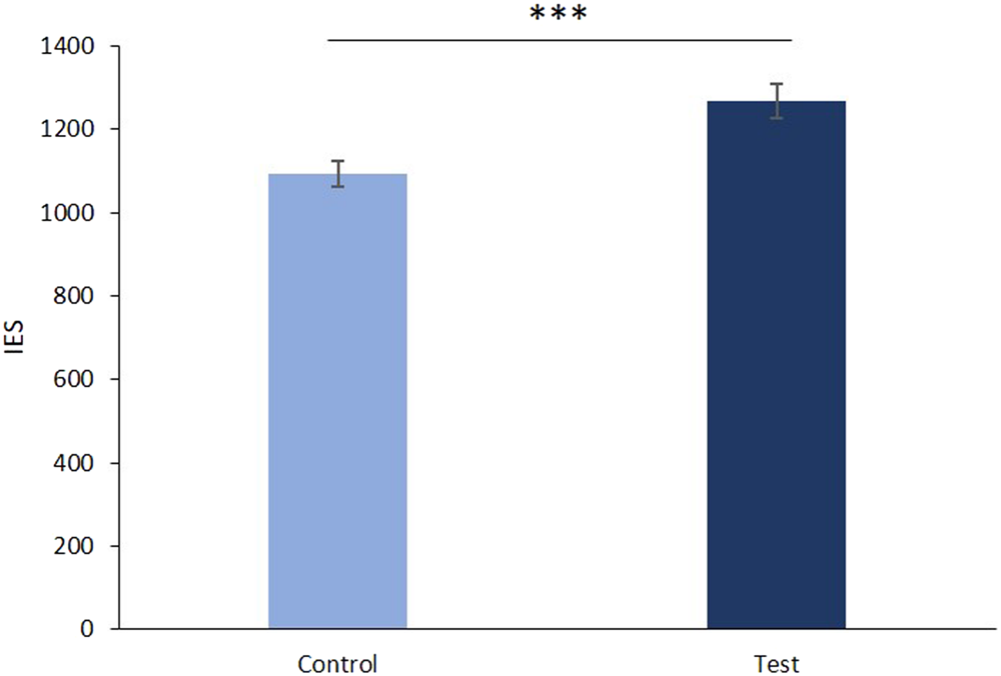
Mean IES in the two types of primes (test vs. control) in the decimal comparison NP task. Error bars depict standard error of the mean. ***p < 0.001.

On the prime, a 2 (Condition: control vs. test) × 2 (Age: Adolescent vs. Adult) × 2 (Gender: Female vs. Male) analysis of variance (ANOVA) revealed a significant main effect of condition *F*(1, 95) = 56.26, *p* < 0.001, η_p_^2^ = 0.37. The main effect of age group was also significant, *F*(1, 95) = 72.93, *p* < 0.001, η_p_^2^ = 0.43. However, the main effect of gender was not significant, *F* < 1. The interaction between the condition and age was significant, *F*(1, 95) = 6.65, *p* = 0.01, η_p_^2^ = 0.04. To control that the interaction was not driven by the overall difference in IES between adults and adolescents, we added the average IES on the prime as a covariate in the ANOVA. This additional analysis revealed no significant condition by age interaction effect, *F* < 1. Thus, both adults and adolescents, regardless of gender, were less efficient at comparing the magnitude of two decimal numbers in which the smallest decimal had the greatest number of digits after the decimal point (e.g., 0.6 vs. 0.131) than at comparing the magnitude of two decimal numbers with the same decimal place (e.g., 0.612 vs. 0.157, see Table 1 and Fig. 3).

**Table 1:** Reaction times (ms), accuracy rates (%) and IES in the two types of prime (test vs. control) in adolescents and adults in the decimal comparison NP task.

	RT		Accuracy		IES	
	Control	Test	Control	Test	Control	Test
Adolescents	1280 (300)	1434 (345)	98.9 (1.7)	94.9 (7.0)	1294 (299)	1522 (401)
Adults	870 (147)	937 (164)	98.8 (1.5)	94.1 (4.9)	881 (147)	998 (184)
All Groups	1082 (314)	1193 (369)	98.8 (1.6)	94.5 (6.0)	1094 (315)	1268 (409)

Standard deviations appear in parentheses.

On the probe, a 2 (Block: Line vs. Luminance) × 2 (Condition: control vs. test) × 2 (Age: Adolescents vs. Adults) × 2 (Gender: Female vs. Male) analysis of variance (ANOVA) revealed a main effect of age, *F*(1, 95) = 90.27, *p* < 0.001, η_p_^2^ = 0.48 and gender, *F*(1, 95) = 14.30, *p* < 0.001, η_p_^2^ = 0.13, but no main effect of block, *F*(1, 95) = 2.74, *p* = 0.10, or condition, *F* < 1. We found a significant interaction between block and condition, *F*(1, 95) = 6.56, *p* = 0.01, η_p_^2^ = 0.06. Lastly, the interactions between block, condition and age (*F* < 1), block, condition and gender (*F* < 1) and block, condition, age and gender (*F* < 1) were not significant.

To further examine the significant interaction between block and condition, we conducted separate ANOVAs for each block.

**Figure 4:**
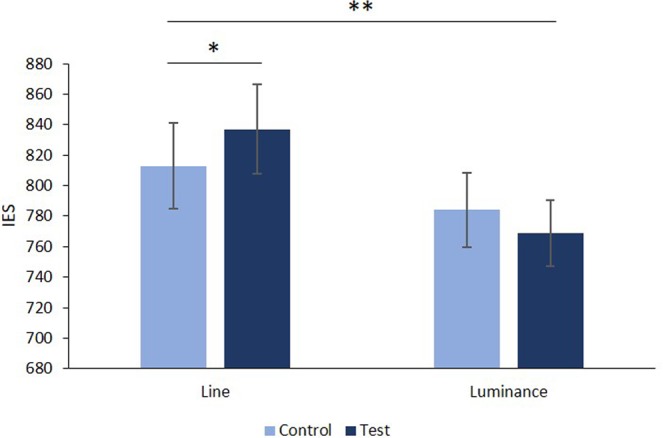
Mean IES for the two types of probes (test vs. control) in adolescents and adults, in the decimal comparison NP task. Error bars depict one standard error of the mean. *p < 0.05, **p < 0.01.

For the Line block, a 2 (Condition: control vs. test) × 2 (Age: Adolescents vs. Adult) × 2 (Sex: Female vs. Male) repeated measures ANOVA revealed a negative priming effect with a significant main effect of condition, *F*(1, 95) = 5.19, *p* = 0.02, η_p_^2^ = 0.05 (see Table 2 and Fig. 4). Participants were less efficient in comparing the length of two lines after comparing a pair of decimal numbers in which the smallest had the greatest number of digits after the decimal point (e.g., 0.6 vs. 0.131) than after comparing a pair of digits with the same number of decimal places (e.g., 0.612 vs. 0.157), i.e., a typical NP effect, see Fig. 4. The main effect of age group, *F*(1, 95) = 85.75, *p* < 0.001, η_p_^2^ = 0.47, and gender, *F*(1, 95) = 12.31, *p* = 0.001, η_p_^2^ = 0.11 was also significant. We found no significant interaction between the condition and age, *F*(1, 95) = 2.17, *p* = 0.14 (F < 1 when the average IES on the probe was entered as a covariate), condition and sex, *F* < 1, or condition, age and sex, *F* < 1.

**Table 2:** Reaction times (ms), Accuracies (%) and IES for the two types of probes (test vs. control) in adolescents and adults, in the decimal comparison NP task.

		RT		Accuracy		IES	
		Control	Test	Control	Test	Control	Test
Line Block	Adolescents	981 (254)	1022 (264)	98.7 (2.3)	99.0 (2.1)	995 (259)	1033 (271)
	Adults	611 (117)	622 (115)	98.8 (2.7)	99.1 (2.3)	619 (120)	628 (118)
	All Groups	802 (272)	828 (287)	98.7 (2.5)	99.9 (2.2)	813 (277)	837 (293)
Luminance Block	Adolescents	891 (214)	883 (175)	98.5 (3.0)	98.4 (3.0)	905 (218)	897 (177)
	Adults	647 (184)	629 (158)	99.3 (1.6)	99.6 (1.3)	653 (191)	631 (158)
	All Groups	773 (234)	760 (209)	98.9 (2.4)	99.0 (2.4)	783 (240)	768 (214)

Standard deviations appear in parentheses.

For the Luminance block, a 2 (Condition: control vs. test) × 2 (Age: Adolescents vs. Adult) × 2 (Gender: Female vs. Male) repeated measures ANOVA revealed a main effect of age, *F*(1, 95) = 38.72, *p* < 0.001, η_p_^2^ = 0.29, and of gender, *F*(1, 95) = 7.10, *p* = 0.009, η_p_^2^ = 0.07, but no main effect of condition, F(1, 95) = 1.68, *p* = 0.19. We found no significant interaction between condition and age, *F* < 1, condition and gender, *F* < 1 or condition, age and gender, *F* < 1. Note that we obtained similar patterns of results when we ran ANOVAs on the prime and probe RTs and accuracies separately (see Supplementary Tables S3 to S18).

## Discussion

The current study aimed to determine (a) whether comparing decimal numbers that do not have the same number of decimal places (e.g., 0.6 vs. 0.131) requires one to inhibit the lengths of the numbers to process their magnitude throughout development in both adolescents and adults and (b) whether the inhibition of non-numerical continuous dimensions when comparing decimal numbers is specific to space, consistent with the functional overlap between the areas processing numbers and spatial extent in the IPS, or extends to other non-numerical continuous dimensions such as luminance.

Consistent with previous studies^30,32,34,46–48^, we observed that participants required more time to compare the magnitude of two decimal numbers when the smallest one has the greatest number of digits after the decimal point (e.g., 0.6 vs. 0.131) than when the two decimal numbers have the same number of decimal places (e.g., 0.612 vs. 0.157). Crucially, in the Line block, participants were less efficient at comparing the length of two lines after having compared the magnitude of two decimal numbers where the smallest one had the greatest number of digits after the decimal point (e.g., 0.6 vs. 0.131, a context in which the length of the numbers interferes with the magnitude comparison) than after having compared the magnitude of two decimal numbers with the same number of decimal places, (e.g., 0.612 vs. 0.157, a context in which length neither interferes with nor facilitates the magnitude comparison). Our finding suggests that regardless of the level of mathematical training (as indexed by age and school level), the comparison of the magnitude of two decimal numbers requires to inhibit the length of the numbers when it interferes with their magnitude, such as in a context in which the smallest decimal number has the largest number of digits (e.g., 0.6 vs. 0.131).

Moreover, our study provides an additional understanding of the source of errors in decimal comparison. Most studies have attributed this error to a whole number bias, whereby the participant relies on a property of whole numbers, such as “the greater the number of digits, the greater its magnitude” to compare decimal numbers in which the smallest one has the greatest number of digits after the decimal point (e.g., 0.6 vs. 0.131)^32,35–37^. Our results show that in addition to a whole number bias in decimal comparison, performances in this task are also impacted by a visuospatial bias due to the interference between the magnitude of the numbers to be compared and their physical length. We postulate that difficulty in comparing decimal numbers in which the smallest one has the greatest number of digits after the decimal point (e.g., 0.6 vs. 0.131) would lie partly in the interference of the perceived spatial extent of the decimal number with the processing of its magnitude. However, the representation of the spatial extent induced by the number of digits in the decimal (*i.e*. numerosity) could also interfere with the processing of the decimal’s magnitude. In the context of this study, it is difficult to disentangle both alternatives especially since the length and the numerosity of the decimal numbers are closely intertwined. Although this does not directly affect the general goal of the present study which was to demonstrate that a visuospatial bias occurs when decimal numbers are presented in a canonical way, future studies should determine more precisely the nature of this visuospatial bias. One way to do so would be to vary the length dimension and the numerosity dimension in the prime stimuli independently. That is, in a pair of decimals with different numbers of digits, the decimal with the least decimal digits would be designed to be longer (or the same length) than the decimal with the most digits. Another possibility would be to add a non-symbolic numerosity magnitude comparison block to the NP task. For example, participants would compare the magnitude of two decimals in the prime and the magnitude of two dot arrays in the probe. We posit that such visuospatial bias results from the progressive co-opting of neural circuits dedicated to the processing of non-numerical magnitudes, specifically spatial extent, for numerical cognition. Interestingly, participants’ responses to another continuous dimension of magnitude, luminosity, were not affected by the preceding decimal comparison. These results are in line with previous studies questioning^17,25^ the existence of a shared system for the representation of different dimensions of magnitude as postulated by Walsh in his ATOM model^49^. In particular, our results are consistent with the idea of shared but distinct representational systems with more overlap between some dimensions, such as number and spatial extent, and less overlap with others, such as luminosity^17,23,25^.

One hypothesis is that neuronal recycling mechanisms are at the root of this overlap. Human mathematics would build from foundational concepts (space, time and number) by progressively co-opting cortical areas whose prior organization fits with cultural needs^27^. The Operational Momentum (OM) effect has been interpreted as a good example of neuronal recycling, as it is thought to be the result of the reuse of cortical circuits in the Posterior Superior Parietal Lobe (PSPL). OM describes a systematic estimation bias according to which one overestimates the outcome of simple addition and underestimates the outcome of subtraction problems^50–53^. Knops and colleagues^50,51^ showed that performing subtractions induces a spatial shift toward left-located responses, while performing addition induces a spatial shift toward right-located responses. Importantly, these spatial shifts recruit the same neural network in the PSPL involved in updating spatial information during saccadic eye movements^50^. Thus, mental arithmetic could superimpose on a parietal circuitry originally associated with spatial coding^50^. Similarly, decimal processing could superimpose on parietal circuits dedicated to spatial extent processing. Indeed, the negative priming effect was observed only on the line probe and not on the luminance probe, which suggests that neural circuits for processing non-numerical dimensions of magnitude such as space but not luminance might be co-opted, not only during the acquisition of simple numerical skills but also during more complex ones such as symbolic decimal number processing. Neurons in the parietal cortex originally dedicated to the processing of continuous non-numerical dimensions of magnitude such as length could thus be co-opted to process decimal numbers. Pre-existing properties of the neurons being co-opted could induce such errors as those observed in decimal comparison, and inhibitory control might be a crucial mechanism in correcting errors induced by the neuronal recycling process. This hypothesis is in keeping with findings showing that overcoming systematic errors in reading, specifically mirror generalization errors (confusing ‘b’ for ‘d’ or ‘p’ for ‘q’), relies on the capacity to inhibit the original function (here the mirror generalization process) of the recycled neurons^54–56^.

In contrast with our assumption and previous studies^42,54,57^, the amplitude of the NP effect in our study did not differ between adolescents and adults. Keeping in mind that the amplitude of the NP effect is considered to indicate the ability to effectively inhibit a specific heuristic or misconception in a given context^58,59^, these results suggests that the inhibition of the visuospatial bias might already be highly efficient in 7th graders. Note that inhibitory control efficiency assessed in a seminal inhibition task (i.e., the Color-Word Stroop task, see Supplementary Materials) did not differ between adolescents and adults, which is coherent with the lack of difference in the amplitude of the NP reported in the present study.

The educational implications of our findings are twofold. First, it appears important that teachers be informed of the role played by domain-general processes such as inhibitory control in academic learning^59^ and in mathematics^60–62^ in particular. In the context of decimal number comparison, teachers could misinterpret student’s errors as a misunderstanding of the rule to be applied while these errors may be the consequence of a difficulty to inhibit both the whole number bias and the visuospatial bias.

Second, considering that student’s errors when comparing decimal numbers may be due to failure to inhibit both the whole number bias and the visuospatial bias, pedagogical interventions based entirely on learning the logical mathematical principles governing the comparison of decimal numbers might not be sufficient to overcome these systematic errors. A more effective way to help children overcome systematic errors and difficulties in decimal comparisons would be to provide pedagogical interventions based on meta-executive learning. Meta-executive intervention typically consists in emphasizing both the logico-mathematical principles to use and the misconception and strategies to inhibit to avoid systematic errors when solving a problem. In the meta-executive intervention developed by Houdé and colleagues^63–65^ in the context of a conditional reasoning task, participants were taught to detect the conflict between their misconception (or reasoning bias) and the logical rule to apply and to solve this conflict. The meta-executive intervention involves raising awareness to the conflict by using executive alarms (“beware there is a trap”) and learning to identify and inhibit the misconception by using a “trap-catcher”. Participants learn to inhibit the response produced by their misconceptions by placing a card materializing the response produced by the misconceptions under the hatched part of the “trap-catcher”. This intervention has been found to be more effective in shifting participants’ response from a biased to a logical response than an intervention focusing and raising awareness to the logical rule to apply^64,65^. In the context of learning to compare decimal numbers, a meta-executive intervention would typically emphasize the need to inhibit both the whole number bias and the visuospatial bias, and the need to activate the mathematical principles governing the comparison of decimal numbers (i.e., comparing the fractional part of decimal numbers by comparing the magnitude of the different place values starting from the first one after the decimal point).

## Supplementary information

Supplementary Tables and information

Dataset S1

## Data Availability

The datasets generated during and analyzed during the current study are available in the Supplementary Materials. Additional materials are available from the corresponding author on reasonable request.
