# Supplementary material for: Evidence for a visuospatial bias in decimal number comparison in adolescents and in adults
Source: Sci Rep. 2019 Oct 14;9:14770. doi: 10.1038/s41598-019-51392-6 (PMC6791935; doi:10.1038/s41598-019-51392-6)
Supplement: Supplementary file 1 — Supplementary Tables and information [file 41598_2019_51392_MOESM1_ESM.docx]

*Table S1. Pairs of decimal numbers to compare in the control, test and filler pairs. The numerical distance between the two decimal numbers and their sum is reported for each pair.*

| Pair Type | Decimal number presented on the left | Decimal number presented on the right | Numerical Distance | Sum of the Digits | Pair Type | Decimal number presented on the left | Decimal number presented on the right | Numerical Distance | Sum of the Digits |
| --- | --- | --- | --- | --- | --- | --- | --- | --- | --- |
| Control | 0.9 | 0.4 | 0.5 | 13 | Test | 0.9 | 0.43 | 0.47 | 16 |
| Control | 0.7 | 0.2 | 0.5 | 9 | Test | 0.6 | 0.12 | 0.48 | 9 |
| Control | 0.3 | 0.8 | 0.5 | 11 | Test | 0.29 | 0.7 | 0.41 | 18 |
| Control | 0.1 | 0.6 | 0.5 | 7 | Test | 0.37 | 0.8 | 0.43 | 18 |
| Control | 0.6 | 0.5 | 0.1 | 11 | Test | 0.4 | 0.38 | 0.02 | 15 |
| Control | 0.4 | 0.3 | 0.1 | 7 | Test | 0.2 | 0.14 | 0.06 | 7 |
| Control | 0.8 | 0.9 | 0.1 | 17 | Test | 0.41 | 0.5 | 0.09 | 10 |
| Control | 0.4 | 0.5 | 0.1 | 9 | Test | 0.26 | 0.3 | 0.04 | 11 |
| Control | 0.69 | 0.15 | 0.54 | 21 | Test | 0.66 | 0.128 | 0.532 | 23 |
| Control | 0.84 | 0.38 | 0.46 | 23 | Test | 0.71 | 0.253 | 0.457 | 18 |
| Control | 0.41 | 0.95 | 0.54 | 19 | Test | 0.439 | 0.97 | 0.531 | 32 |
| Control | 0.29 | 0.75 | 0.46 | 23 | Test | 0.361 | 0.82 | 0.459 | 20 |
| Control | 0.87 | 0.73 | 0.14 | 25 | Test | 0.69 | 0.554 | 0.136 | 29 |
| Control | 0.62 | 0.56 | 0.06 | 19 | Test | 0.73 | 0.673 | 0.057 | 26 |
| Control | 0.23 | 0.37 | 0.14 | 15 | Test | 0.821 | 0.96 | 0.139 | 26 |
| Control | 0.66 | 0.72 | 0.06 | 21 | Test | 0.459 | 0.51 | 0.051 | 24 |
| Control | 0.981 | 0.444 | 0.537 | 30 | Test | 0.7 | 0.213 | 0.487 | 13 |
| Control | 0.612 | 0.157 | 0.455 | 22 | Test | 0.9 | 0.476 | 0.424 | 26 |
| Control | 0.329 | 0.862 | 0.533 | 30 | Test | 0.131 | 0.6 | 0.469 | 11 |
| Control | 0.583 | 0.941 | 0.358 | 30 | Test | 0.337 | 0.8 | 0.463 | 21 |
| Control | 0.254 | 0.116 | 0.138 | 19 | Test | 0.6 | 0.521 | 0.079 | 14 |
| Control | 0.936 | 0.871 | 0.065 | 34 | Test | 0.7 | 0.678 | 0.022 | 28 |
| Control | 0.658 | 0.792 | 0.134 | 37 | Test | 0.482 | 0.5 | 0.018 | 19 |
| Control | 0.271 | 0.339 | 0.068 | 25 | Test | 0.714 | 0.8 | 0.086 | 20 |
| Filler | 0.62 | 0.1 | 0.52 | 9 | Filler | 0.67 | 0.73 | 0.06 | 23 |
| Filler | 0.78 | 0.2 | 0.58 | 17 | Filler | 0.826 | 0.3 | 0.526 | 19 |
| Filler | 0.4 | 0.53 | 0.13 | 12 | Filler | 0.751 | 0.2 | 0.551 | 15 |
| Filler | 0.7 | 0.81 | 0.11 | 16 | Filler | 0.5 | 0.697 | 0.197 | 27 |
| Filler | 0.975 | 0.43 | 0.545 | 28 | Filler | 0.8 | 0.932 | 0.132 | 22 |
| Filler | 0.642 | 0.18 | 0.462 | 21 | Filler | 0.25 | 0.394 | 0.144 | 23 |

*Table S2. Average number of accurate trials per condition (control, test or filer), type of trial (Prime vs. Probe) and age group (Adults vs. Adolescents). Notably participants were given 24 trials in total in the control and test conditions in both prime and probe and 12 trials in total in the filer condition in both prime and probe.*

|  | Adult | |  | Adolescent | |
| --- | --- | --- | --- | --- | --- |
|  | Prime | Probe |  | Prime | Probe |
| Control | 23.7 | 23.4 |  | 23.7 | 23.4 |
| Test | 22.6 | 22.3 |  | 22.6 | 22.3 |
| Filer | 11.8 | 11.7 |  | 11.5 | 11.3 |

1. **Decimal Comparison NP Task**
   1. **Prime reaction times analyses**

*Table S3. Within Subject effect of 2 (Condition: control vs. test) x 2 (Age: Adolescent vs. Adult) x 2 (Gender: Female vs. Male) analysis of variance (ANOVA)*

|  | df | F | p |
| --- | --- | --- | --- |
| Condition | 1 | 49.53 | < .001 |
| Condition x Age | 1 | 8.73 | 0.004 |
| Condition x Gender | 1 | 0.20 | 0.655 |
| Condition x Age x Gender | 1 | 0.02 | 0.872 |
| Residuals | 95 |  |  |

*Table S4. Between Subject effect of 2 (Condition: control vs. test) x 2 (Age: Adolescent vs. Adult) x 2 (Gender: Female vs. Male) analysis of variance (ANOVA)*

|  | df | F | p |
| --- | --- | --- | --- |
| Age | 1 | 75.08 | < .001 |
| Gender | 1 | 0.22 | 0.639 |
| Age x Gender | 1 | 1.30 | 0.256 |
| Residuals | 95 |  |  |

- 1. **Prime accuracy rates analyses**

*Table S5. Within Subject effect of 2 (Condition: control vs. test) x 2 (Age: Adolescent vs. Adult) x 2 (Gender: Female vs. Male) analysis of variance (ANOVA)*

|  | df | F | p |
| --- | --- | --- | --- |
| Condition | 1 | 46.43 | < .001 |
| Condition x Age | 1 | 0.20 | 0.651 |
| Condition x Gender | 1 | 0.03 | 0.846 |
| Condition x Age x Gender | 1 | 0.31 | 0.573 |
| Residuals | 95 |  |  |

*Table S6. Between Subject effect of 2 (Condition: control vs. test) x 2 (Age: Adolescent vs. Adult) x 2 (Gender: Female vs. Male) analysis of variance (ANOVA)*

|  | df | F | p |
| --- | --- | --- | --- |
| Age | 1 | 0.30 | 0.580 |
| Gender | 1 | 0.04 | 0.826 |
| Age x Gender | 1 | 0.35 | 0.554 |
| Residuals | 95 |  |  |

- 1. **Probe reaction times analyses**

*Table S7. Within Subject effect of 2 (Block: Line vs. Luminance) x 2 (Condition: control vs. test) x 2 (Age: Adolescent vs. Adult) x 2 (Gender: Female vs. Male) analysis of variance (ANOVA)*

|  | df | F | p |
| --- | --- | --- | --- |
| Condition | 1 | 0.39 | 0.531 |
| Condition x Age | 1 | 0.03 | 0.861 |
| Condition x Gender | 1 | 0.09 | 0.763 |
| Condition x Age x Gender | 1 | 1.44 | 0.233 |
| Block | 1 | 0.30 | 0.583 |
| Block x Age | 1 | 3.05 | 0.084 |
| Block x Gender | 1 | 0.08 | 0.778 |
| Block x Age x Gender | 1 | 1.30 | 0.257 |
| Condition x Block | 1 | 0.10 | 0.750 |
| Condition x Block x Age | 1 | 0.14 | 0.703 |
| Condition x Block x Gender | 1 | 0.50 | 0.480 |
| Condition x Block x Age x Gender | 1 | 1.85 | 0.176 |
| Residuals | 95 |  |  |

*Table S8. Between Subject effect of 2 (Block: Line vs. Luminance) x 2 (Block: control vs. test) x 2 (Age: Adolescent vs. Adult) x 2 (Gender: Female vs. Male) analysis of variance (ANOVA)*

|  | df | F | p |
| --- | --- | --- | --- |
| Age | 1 | 1.80 | 0.182 |
| Gender | 1 | 3.10 | 0.081 |
| Age x Gender | 1 | 0.06 | 0.805 |
| Residuals | 95 |  |  |

- - 1. **Line Block: Probe reaction times analyses**

*Table S9. Within Subject effect of 2 (Condition: control vs. test) x 2 (Age: Adolescent vs. Adult) x 2 (Gender: Female vs. Male) analysis of variance (ANOVA)*

|  | df | F | p |
| --- | --- | --- | --- |
| Condition | 1 | 6.58 | 0.012 |
| Condition x Age | 1 | 2.71 | 0.103 |
| Condition x Gender | 1 | 0.06 | 0.803 |
| Condition x Age x Gender | 1 | 0.09 | 0.758 |
| Residuals | 95 |  |  |

*Table S10. Between Subject effect of 2 (Condition: control vs. test) x 2 (Age: Adolescent vs. Adult) x 2 (Gender: Female vs. Male) analysis of variance (ANOVA)*

|  | df | F | p |
| --- | --- | --- | --- |
| Age | 1 | 87.27 | < .001 |
| Gender | 1 | 11.63 | < .001 |
| Age x Gender | 1 | 5.91 | 0.017 |
| Residuals | 95 |  |  |

- - 1. **Luminance Block: Probe reaction times analyses**

*Table S11. Within Subject effect of 2 (Condition: control vs. test) x 2 (Age: Adolescent vs. Adult) x 2 (Gender: Female vs. Male) analysis of variance (ANOVA)*

|  | Df | F | p |
| --- | --- | --- | --- |
| Condition | 1 | 1.47 | 0.227 |
| Condition x Age | 1 | 0.17 | 0.673 |
| Condition x Gender | 1 | 0.06 | 0.795 |
| Condition x Age x Gender | 1 | 0.09 | 0.756 |
| Residuals | 95 |  |  |

*Table S12. Between Subject effect of 2 (Condition: control vs. test) x 2 (Age: Adolescent vs. Adult) x 2 (Gender: Female vs. Male) analysis of variance (ANOVA)*

|  | Df | F | p |
| --- | --- | --- | --- |
| Age | 1 | 36.75 | < .001 |
| Gender | 1 | 6.61 | 0.012 |
| Age x Gender | 1 | 0.02 | 0.864 |
| Residuals | 95 |  |  |

- 1. **Probe accuracy rates analyses**

*Table S13. Within Subject effect of 2 (Block: Line vs. Luminance) x 2 (Condition: control vs. test) x 2 (Age: Adolescent vs. Adult) x 2 (Gender: Female vs. Male) analysis of variance (ANOVA)*

|  | Df | F | p |
| --- | --- | --- | --- |
| Condition | 1 | 0.46 | 0.495 |
| Condition x Age | 1 | 1.74 | 0.190 |
| Condition x Gender | 1 | 0.01 | 0.970 |
| Condition x Age x Gender | 1 | 0.17 | 0.677 |
| Block | 1 | 2.81 | 0.097 |
| Block x Age | 1 | 11.04 | 0.001 |
| Block x Gender | 1 | 0.81 | 0.370 |
| Block x Age x Gender | 1 | 5.71 | 0.019 |
| Condition x Block | 1 | 7.35 | 0.008 |
| Condition x Block x Age | 1 | 0.59 | 0.443 |
| Condition x Block x Gender | 1 | 0.14 | 0.706 |
| Condition x Block x Age x Gender | 1 | 0.01 | 0.966 |
| Residuals | 95 |  |  |

*Table S14. Between Subject effect of 2 (Block: Line vs. Luminance) x 2 (Block: control vs. test) x 2 (Age: Adolescent vs. Adult) x 2 (Gender: Female vs. Male) analysis of variance (ANOVA)*

|  | df | F | p |
| --- | --- | --- | --- |
| Age | 1 | 86.94 | < .001 |
| Gender | 1 | 13.06 | < .001 |
| Age x Gender | 1 | 1.96 | 0.164 |
| Residuals | 95 |  |  |

- - 1. **Line Block: Probe accuracy rates analyses**

*Table S15. Within Subject effect of 2 (Condition: control vs. test) x 2 (Age: Adolescent vs. Adult) x 2 (Gender: Female vs. Male) analysis of variance (ANOVA)*

|  | df | F | p |
| --- | --- | --- | --- |
| Condition | 1 | 0.46 | 0.498 |
| Condition x Age | 1 | 0.02 | 0.886 |
| Condition x Gender | 1 | 0.07 | 0.779 |
| Condition x Age x Gender | 1 | 3.35 | 0.070 |
| Residuals | 95 |  |  |

*Table S16. Between Subject effect of 2 (Condition: control vs. test) x 2 (Age: Adolescent vs. Adult) x 2 (Gender: Female vs. Male) analysis of variance (ANOVA)*

|  | df | F | p |
| --- | --- | --- | --- |
| Age | 1 | 0.05 | 0.811 |
| Gender | 1 | 2.16 | 0.144 |
| Age x Gender | 1 | 0.37 | 0.541 |
| Residuals | 95 |  |  |

- - 1. **Luminance Block: Probe accuracy rates analyses**

*Table S17. Within Subject effect of 2 (Condition: control vs. test) x 2 (Age: Adolescent vs. Adult) x 2 (Gender: Female vs. Male) analysis of variance (ANOVA)*

|  | df | F | p |
| --- | --- | --- | --- |
| Condition | 1 | 0.05 | 0.823 |
| Condition x Age | 1 | 0.15 | 0.699 |
| Condition x Gender | 1 | 0.49 | 0.483 |
| Condition x Age x Gender | 1 | 0.01 | 0.928 |
| Residuals | 95 |  |  |

*Table S18. Between Subject effect of 2 (Condition: control vs. test) x 2 (Age: Adolescent vs. Adult) x 2 (Gender: Female vs. Male) analysis of variance (ANOVA)*

|  | df | F | p |
| --- | --- | --- | --- |
| Age | 1 | 4.72 | 0.032 |
| Gender | 1 | 1.15 | 0.286 |
| Age x Gender | 1 | 0.93 | 0.337 |
| Residuals | 95 |  |  |

1. **Color-Word Stroop task**
   1. **Materials and procedures**

Sixteen items were created by combining four words denoting colors (red, green, blue and yellow) with four corresponding ink color (RGB color codes were 255;0;0 for red, 0;255;0 for green, 0;0;255 for blue, and 255;255;0 for yellow). Combining the four words and the four ink colors produced four congruent items in which the color denoted by the word was congruent with the color of the ink (e.g., ‘red’ in red) and 12 incongruent items in which the color denoted by the word was incongruent with the color of the ink (e.g., ‘red’ in blue). The words were presented at the center of the screen in 24-pt Courier New Bold type (1° of visual angle) on a gray background (RGB code 135;135;135).

Participants were instructed to identify the ink colors while ignoring the meaning of the words presented. Participants responded by manually pressing two keyboard buttons with their left and right index fingers. Participants were to press the ‘s’ button for yellow and blue and the ‘l’ button for red and green (color chips of the respective colors were added to the keyboard buttons to simplify responses).

As shown in Supplementary Figure 1, each trial began with a fixation cross (1500 ms) followed by a word that was displayed until the participant responded (with a time limit of 3000 ms).

Participants performed an experimental block of 64 trials in which half of the items were congruent (e.g., RED written in red) and half were incongruent (e.g., RED written in blue). The order of presentation of the trials was pseudo-randomized, with no more than four congruent or four incongruent stimuli occurring in a row. The experimental block was preceded by two training blocks of 16 randomly ordered trials in which participants received simple feedback on accuracy (correct/incorrect). In the first training block, participants had to determine the color of a dot presented in the center of the screen by pressing on the correct response button (each of the four colors was presented four times). The second training block consisted of eight congruent and eight incongruent trials presented in a random order.


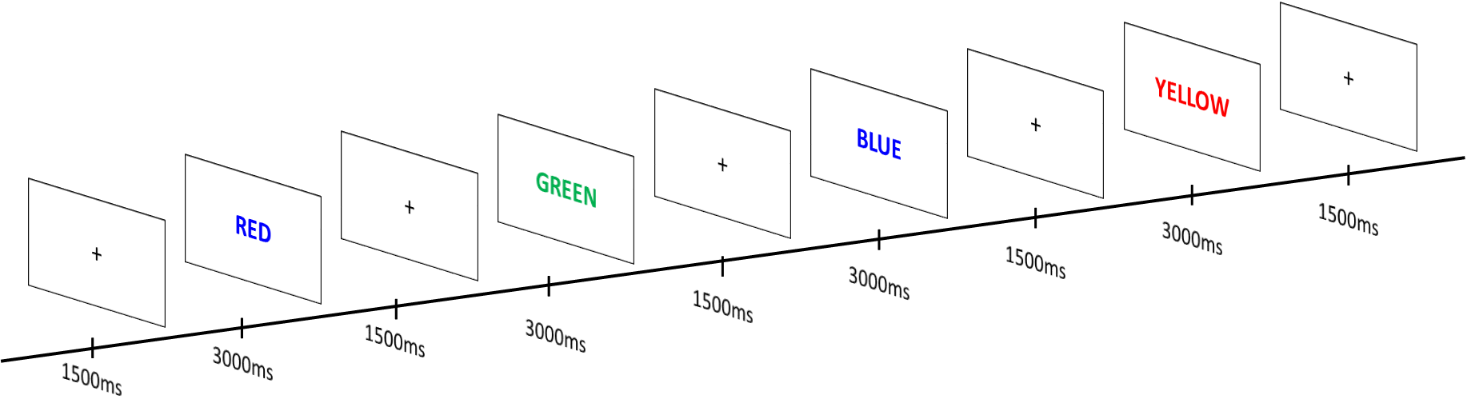
Supplementary Figure 1. Example of trials in the Color-Word Stroop task.

- 1. **Inverse efficiency analysis**

Only correct trials were taken into account for the analyses of RTs, while for each participant in each condition, RTs +/- 2 SD from the individual mean for a given condition were deleted. As a result, we removed on average 1% (± 2.49%) and 1% (± 0.94%) of the RTs for adolescents and adults respectively. Note that two adolescents were excluded from the Color-Word Stroop task analysis, as they performed at chance level. Additionally, one adult participant was not able to finish the task. For each participant, we computed the IES for both the incongruent and congruent items. Our data complied with the recommendations of Bruyer and Brysbaert (2011): accuracy was high (i.e., mean greater than 97%), accuracies and RTs went in the same direction and we observed no speed-accuracy trade-off (*r*s < .19, *p*s *>* .05).

A 2 (Type of Item: Incongruent vs. Congruent) x 2 (Age: Adolescents vs. Adults) x 2 (Gender: Female vs. Male) ANOVA on IES revealed a significant main effect of the type of item, *F*(1, 93) = 19.92, *p* < .001, η_p_^2^ = .17, with participants being less efficient at identifying the ink color when it was incongruent than congruent with the color denoted by the word (see Table S20). The main effects of age, *F*(1, 93) = 40.12, *p* < .001, η_p_^2^ =.28 and gender, *F*(1, 93) = 4.77, *p* = .03, η_p_^2^ = .03, were also significant. However, we found no significant interaction between type of item and age, *F*(1, 93) = 1.18, *p* = .29 (F < 1 when the average IES was entered as a covariate in the ANOVA), or type of item and gender, *F* < 1 or type of item, age and gender, *F* < 1. Note that we obtained similar patterns of results when we ran ANOVAs on the RTs and accuracies (see Tables S21)

*Table S20. Reaction times (ms), accuracies, (%) and IES for the two types of items (Incongruent* vs. *Congruent) in adolescents and adults in the Color-Word Stroop task. Standard deviations appear in parentheses.*

|  | - RT | |  | - Accuracy | |  | - IES | |
| --- | --- | --- | --- | --- | --- | --- | --- | --- |
|  | - Congruent | - Incongruent |  | - Congruent | - Incongruent |  | - Congruent | - Incongruent |
| - Adolescents | 961 (159) | - 1040 (217) |  | - 96.6 (8.2) | - 96.3 (8.4) |  | - 1010 (242) | - 1099 (319) |
| - Adults | - 694 (149) | 746 (160) |  | - 98.5 (3.2) | - 97.5 (4.1) |  | - 706 (162) | - 767 (174) |
| - All Groups | - 832 (204) | - 897 (241) |  | - 97.5 (6.4) | - 96.9 (6.7) |  | - 863 (257) | - 938 (307) |

- 1. **Reaction times analyses**

*Table S18. Within Subject effect of 2 (Type of Item: Incongruent vs. Congruent) x 2 (Age: Adolescent vs. Adult) x 2 (Gender: Female vs. Male) analysis of variance (ANOVA)*

|  | df | F | p |
| --- | --- | --- | --- |
| Type of item | 1 | 23.21 | < .001 |
| Type of item x Age | 1 | 1.20 | 0.275 |
| Type of item x Gender | 1 | 0.25 | 0.613 |
| Type of item x Age x Gender | 1 | 0.10 | 0.752 |
| Residuals | 95 |  |  |

*Table S19. Between Subject effect of 2 (Type of Item: Incongruent vs. Congruent) x 2 (Age: Adolescent vs. Adult) x 2 (Gender: Female vs. Male) analysis of variance (ANOVA)*

|  | df | F | p |
| --- | --- | --- | --- |
| Age | 1 | 58.02 | < .001 |
| Gender | 1 | 4.93 | 0.029 |
| Age x Gender | 1 | 0.55 | 0.459 |
| Residuals | 95 |  |  |

- 1. **Accuracy rates analyses**

*Table S21. Within Subject effect of 2 (Type of Item: Incongruent vs. Congruent) x 2 (Age: Adolescent vs. Adult) x 2 (Gender: Female vs. Male) analysis of variance (ANOVA)*

|  | df | F | p |
| --- | --- | --- | --- |
| Type of item | 1 | 0.47 | 0.492 |
| Type of item x Age | 1 | 0.01 | 0.924 |
| Type of item x Gender | 1 | 3.74 | 0.056 |
| Type of item x Age x Gender | 1 | 0.32 | 0.568 |
| Residuals | 95 |  |  |

*Table S21. Between Subject effect of 2 (Type of Item: Incongruent vs. Congruent) x 2 (Age: Adolescent vs. Adult) x 2 (Gender: Female vs. Male) analysis of variance (ANOVA)*

|  | df | F | p |
| --- | --- | --- | --- |
| Age | 1 | 1.215 | 0.273 |
| Gender | 1 | 0.813 | 0.370 |
| Age x Gender | 1 | 0.957 | 0.330 |
| Residuals | 95 |  |  |
